# Micronized Copper Wood Preservatives: Efficacy of Ion, Nano, and Bulk Copper against the Brown Rot Fungus *Rhodonia placenta*

**DOI:** 10.1371/journal.pone.0142578

**Published:** 2015-11-10

**Authors:** Chiara Civardi, Mark Schubert, Angelika Fey, Peter Wick, Francis W. M. R. Schwarze

**Affiliations:** 1 Empa, Applied Wood Materials, Dübendorf, St. Gallen, Switzerland; 2 ETH, Institute for Building Materials, Zürich, Switzerland; 3 Empa, Particles- Biology Interactions, St. Gallen, Switzerland; Universita degli Studi di Pisa, ITALY

## Abstract

Recently introduced micronized copper (MC) formulations, consisting of a nanosized fraction of basic copper (Cu) carbonate (CuCO_3_·Cu(OH)_2_) nanoparticles (NPs), were introduced to the market for wood protection. Cu NPs may presumably be more effective against wood-destroying fungi than bulk or ionic Cu compounds. In particular, Cu- tolerant wood-destroying fungi may not recognize NPs, which may penetrate into fungal cell walls and membranes and exert their impact. The objective of this study was to assess if MC wood preservative formulations have a superior efficacy against Cu-tolerant wood-destroying fungi due to nano effects than conventional Cu biocides. After screening a range of wood-destroying fungi for their resistance to Cu, we investigated fungal growth of the Cu-tolerant fungus *Rhodonia placenta* in solid and liquid media and on wood treated with MC azole (MCA). In liquid cultures we evaluated the fungal response to ion, nano and bulk Cu distinguishing the ionic and particle effects by means of the Cu^2+^ chelator ammonium tetrathiomolybdate (TTM) and measuring fungal biomass, oxalic acid production and laccase activity of *R*. *placenta*. Our results do not support the presence of particular nano effects of MCA against *R*. *placenta* that would account for an increased antifungal efficacy, but provide evidence that attribute the main effectiveness of MCA to azoles.

## Introduction

Copper (Cu) has long been known for its fungicidal properties and it is an essential biocide for wood in contact with the soil, as it is the only active substance that hitherto successfully inhibits wood decomposition by soft rot fungi [1]. The efficacy of Cu- based wood preservatives against wood- destroying fungi is mainly exerted by Cu in its soluble form, as Cu^2+^ ions [2–4]. However, an increased copper efficacy may be achieved at the nanoscale: several nanoparticles (NPs) have been shown to be more toxic to prokaryotes and eukaryotes than larger particles of the same chemical composition [5, 6] or dissolved ions [7–9]. In fungi, it is believed that NPs may enter the fungal cell through endocytosis [10] or, if smaller than the pores, across the cell walls eluding barrier and entering the plasma membrane [11, 12]. Afterwards, the NPs may be able to cross the cell membrane, enter into the fungal cell and get in direct contact with cell components. However, to date there is a lack of understanding of their specific mode of action in fungi, despite their key role in wood decomposition of treated wood or bioremediation of contaminated soils. Recently, basic CuCO_3_·Cu(OH)_2_ particulate systems for wood protection were introduced to the US market [13]. These wood preservatives, commonly known as micronized copper (MC) formulations, contain a considerable amount of nanosized CuCO_3_·Cu(OH)_2_ with low water solubility, and in some cases purely consist of nanoparticles [14].

When wood is treated with MC, some Cu reacts with wood and part of it remains in the form of unreacted Cu particles that provide a reservoir effect [15]. Most of the studies [16] on Cu bioavailability of micronized Cu have focused on the release of Cu^2+^ ions [17–21]. However, the superior efficacy of the treatment [22–25] may be partially due to insolubilized persistent CuCO_3_·Cu(OH)_2_ NPs that diffuse through the fungal cell wall and its membrane and exert their toxic effects, also described as the Trojan horse mechanism [26].

In Cu- tolerant (brown rot) fungi the threshold for Cu- NPs may be lower than the threshold for Cu^2+^ ions, due to nano formulation, and subsequently fungi may not be able to trigger Cu- tolerance mechanisms in presence of small amounts of MC. In this case, the main wood degradation mechanisms for brown rot fungi, i.e. free hydroxyl radical production via Fenton reaction, may be impaired. Thus, the production of mediators for the Fenton reaction, which also implicates Cu oxidases [27, 28], may not be stimulated by Cu- tolerant fungi, resulting in a biochemical activity pattern dissimilar from the array that occurs when Cu- tolerant fungi are exposed to bulk or ionic Cu. In particular, the production of oxalic acid [29] or laccase [30] may be reduced. Although Tang et al. [27] provided a thorough investigation on the gene expression of fungi exposed to MC, so far no comparison with conventional Cu-based wood preservatives has been made.

Therefore the objective of this study was to determine if MC is more effective than standard Cu compounds against wood-destroying fungi due to specific nano formulation. For this purpose we first investigated the growth of different wood-destroying fungi in different MC-amended solid media and selected the most Cu-tolerant strain for further investigation. Subsequently, we assessed growth and enzyme activities of the selected Cu- tolerant fungus *Rhodonia placenta* (Fr.) Niemelä, Larss. & Schigel *(= P*. *placenta)* in liquid cultures amended with Cu^2+^ ions from Cu sulfate (CuSO_4_), MC (nano), bulk CuCO_3_·Cu(OH)_2_, with and without ammonium tetrathiomolybdate (TTM) a well-known Cu^2+^ ion chelator used to treat Cu poisoning and Wilson’s disease in human and animals [31–34]. In fungi, a high dose of TTM (IC_50_ 1.0±0.2 μM) can inhibit the production of tyrosinase [35], an enzyme responsible for melanin synthesis, but low dosages are well tolerated. The ligand binds selectively to Cu^2+^ ions, allowing us to investigate whether Cu^2+^ ions derived from solubilized Cu-NPs and is responsible for the toxicity [36] in Cu- amended fungal culture media. The impact of MC co-biocide, tebuconazole (TBA), was also assessed.

Two mediators involved in the Fenton reaction were measured to assess different fungal responses to Cu: oxalic acid and laccase. Oxalic acid is believed to be a key component in Cu detoxification due to its ability to bind Cu, whereas laccase is produced by *R*. *placenta* during wood colonization [29]. Subsequently, wood decay caused by *R*. *placenta* on wood samples treated with MC, bulk CuCO_3_·Cu(OH)_2_ and TBA was assessed according to EN 113 [37] to determine the effectiveness of the different components in a more natural setting.

## Materials and Methods

### Materials

2,2-azino-bis(3-ethylbenzothiazoline-6-sulfonic acid) (ABTS), bulk CuCO_3_·Cu(OH)_2_, CuSO_4_, TBA and TTM were purchased from Sigma Aldrich, while agar, malt extract and potassium chloride from VWR (Oxoid, Darmstadt, Germany).

The oxalic acid assay Enzytec oxalic acid was purchased from R-Biopharm AG. The silver stain kit, Dodeca Silver Stain was obtained from BIO-RAD.

Two commercial aqueous suspensions of MCA were investigated. The two MCA formulations contain comparable amounts of Cu particles but differ in the amount of TBA: MCA_HTBA contained 5% w/w and MCA_LTBA 0.4% w/w TBA.

#### NP characterization

Cu particles in the MCA formulations were characterized prior to fungal exposure. Particle morphology was assessed by transmission electron microscopy (TEM) with a Zeiss 900 microscope (Zeiss SMT, Oberkochen, Germany). TEM grids (400 mesh) coated with 8 nm of carbon were incubated for 20 s on a 10 μl droplet of MCA diluted with nanopure water. The excess suspension fluid was drawn off with filter paper.

Particle size distribution was measured by nanoparticle tracking analysis (NTA) using a NanoSight LM20 (NanoSight Ltd., UK) on MCA diluted with Milli-Q water. Data analysis was performed with NTA 2.3.5 software (NanoSight Ltd., UK). Particle diameters are reported as average and standard deviation of seven video recording of the sample. Zeta potential measurements were carried out on MCA diluted with Milli-Q water using a Zetasizer NanoZS (Malvern Instruments, UK).

### Screening for Cu-tolerance

All fungi used in the solid media study were wood-destroying basidiomycetes commonly used in EN 113 tests: *Antrodia serialis* (Fr.) Donk isolate 43, *Coniophora puteana* (Schumach.) Karst isolate 62, *Gloeophyllum trabeum* (Pers.) Murrill isolate 100, *R*. *placenta* isolate 45, *Trametes versicolor* (L.) Lloyd isolate 159 from the Empa culture collection. Fungal mycelia (9 mm in diameter) were grown in 9 cm Petri dishes with 25 mL solid medium (autoclave sterilized) containing 4% (w/v) malt extract and 2.5% (w/v) agar. The media were amended with either 0.01% (w/v) or 0.05% (w/v) of MCA_HTBA or MCA_LTBA. Three replicates were prepared for each condition. Cultures were stored at 22°C and 70% RH. The cultures were inspected regularly, and their 4 cardinal points were marked to determine the growth radii until the colonies reached the edges of the Petri dishes. Fungal growth rate for each colony (in mm per day) was determined dividing the mean value of the latest growth radii minus that of the earliest by the number of days elapsed between the measurements.

### Fungal response to Cu^2+^ ions and particles

The fungus used in the liquid culture study was *R*. *placenta* isolate 45 from the Empa culture collection. Fungal mycelia were grown in 500 mL Erlenmeyer flasks with 250 mL liquid culture medium (autoclave sterilized) containing 1% (w/v) malt extract and 0.6% (w/v) potassium chloride.

To understand the effect of ion, nano or bulk Cu, the following materials were added respectively: CuSO_4_, MCA, CuCO_3_·Cu(OH)_2_. The concentration of CuCO_3_·Cu(OH)_2_, and CuSO_4_ was 0.02 mM. The quantity of MCA was calculated based on the equivalent 0.02 mM of total Cu. The interference caused by the azole biocide on the fungus was assessed by adding TBA. The amount of TBA, alone or with Cu, was calculated as the amount of TBA content in MCA (5% w/w). TTM (0.02 mM) was used to separate the Cu-based particles from the Cu^2+^ ions. All these materials were added to the liquid cultures as indicated in Table 1.

**Table 1:** Scheme of the liquid cultures used. TTM = ammonium tetrathiomolybdate; MCA = micronized copper azole; CuCO_3_ = Cu carbonate; TBA = tebuconazole; CuSO_4_ = Cu sulfate.

Liquid culture media	
Without TTM	With 0.02 mM TTM
Control	Control
0.02 mM MCA	0.02 mM MCA
0.02 mM CuCO_3_·Cu(OH)_2_	0.02 mM CuCO_3_·Cu(OH)_2_
0.02 mM CuCO_3_·Cu(OH)_2_ + 5% w/w TBA	0.02 mM CuCO_3_·Cu(OH)_2_ + 5% w/w TBA
0.02 mM CuSO_4_	0.02 mM CuSO_4_
5% w/w TBA	---

Each treatment was repeated in triplicates. Each flask was inoculated with 1 disc (8 mm in diameter) of the strain pre-cultured in solid medium and incubated in an orbital shaker (100 rpm) at 22°C for 9 weeks. The pH of the liquid culture was between 4.5 and 5, assuming that some Cu particles would not solubilize but would remain suspended in the liquid media. After incubation, the biomass was harvested by filtration and oven dried at 107°C for 24 hours. Fungal growth was estimated as wet and dry biomass weight.

#### Laccase activity

Laccase activity in *R*. *placenta* 45 was measured after 16 weeks incubation in untreated, MCA_LTBA and MCA_HTBA-treated wood colonized by *R*. *placenta* 45 (see protective effectiveness of MCA active ingredients) and after 2, 4, and 9 weeks in liquid cultures. For detection of laccase in wood, the colonized samples were treated according to Wei et al. [30]. The grounded wood was stirred overnight at 4C° in dist. H_2_O containing 1M NaCl to extract extracellular proteins. The liquid phase was separated from the solids by vacuum filtration through Whatman no 1 paper and concentrated in an ultrafiltration cell (Ultracel, Millipore) fitted with 10-kDa cutoff membrane.

Laccase activity was measured as initial velocity of the oxidation of ABTS (2,2'-azino-bis(3-ethylbenzothiazoline-6-sulphonic acid), 3 mM) to its cation radical at room temperature (22–25°C) and at pH 4.5 (citrate buffer 100 mM). Changes in absorbance (*Δ*A) at 420 nm were recorded with UV-visible spectrophotometer (Genesys 10S UV-vis, Thermo Scientific Inc., Waltham, MA, USA). One volumetric activity unit (U) was defined as the amount of enzyme transforming 1 μmol of ABTS per min and the volumetric activities were calculated using an extinction coefficient (ε) of 36000 mol^-1^ L cm^-1^[38, 39].

#### Oxalic acid assay

After incubation, 0.5 mL of liquid media were taken from each culture medium and oxalate concentration in the samples was analyzed with a spectrophotometer (Genesys 10S UV-vis, Thermo Scientific Inc., Waltham, MA, USA) at 590 nm as specified by the instruction manual (Enzytec Oxalic acid, R-Biopharm AG). Samples were diluted 100-fold with distilled water before being used in the assay as they showed very high activities.

To determine any further changes in the protein secretions of *R*. *placenta* 45 exposed to the different amended media after incubation, 0.2 mL of liquid media was taken from each culture medium. Protein extracts from the control, CuCO_3_·Cu(OH)_2_, CuSO_4_, MCA liquid media -with and without TTM- plus markers (BIO-RAD) were separated by sodium dodecyl sulfate-polyacrylamide gel electrophoresis (SDS-PAGE) using 10% gels loaded with 5 μL of each liquid culture sample and the marker. Gels were silver stained with the Dodeca Silver Stain kit according to the instruction manual (BIO-RAD).

### Efficacy of MCA active substances

Scots pine (*Pinus sylvestris L*.) sapwood blocks (50 x 25 x 15 mm) were pressure treated according to the European standard EN 113 [37] with different concentrations of MCA (2%, 1.6%, 1.33%, 1.07%, 0.8%, 0%). Scots pine sapwood blocks (50 x 25 x 15 mm) were also impregnated with 2% and 1.6% equivalent concentrations of CuCO_3_·Cu(OH)_2_ or TBA. No permits were required for the described study, which complied with all relevant regulations. No endangered or protected species were involved. After drying, the samples were exposed to *R*. *placenta* 45 at 22°C and 70% RH. Test procedures were performed according to the European standard EN 113 [37]. After incubation, wood blocks were removed from the culture vessels, brushed free of mycelium and oven dried at 103±1°C. The percentage of weight loss was calculated from the dry weight before and after the test.

### Statistical analysis

Growth data and oxalic acid concentrations from fungi growing on solid and liquid media and on wood were log-transformed and data expressed as percentages, such as mass loss, were arcsine-transformed prior to analysis (ANOVA) and back-transformed to numerical values for visualization. Means were separated using Tukey’s-HSD (Honestly Significant Difference) test at significance level p<0.05. The statistical package used for all analyses was SPSS (Version 17.0, SPSS Inc., Chicago, IL, USA).

## Results

### NP characterization

**Fig 1:**
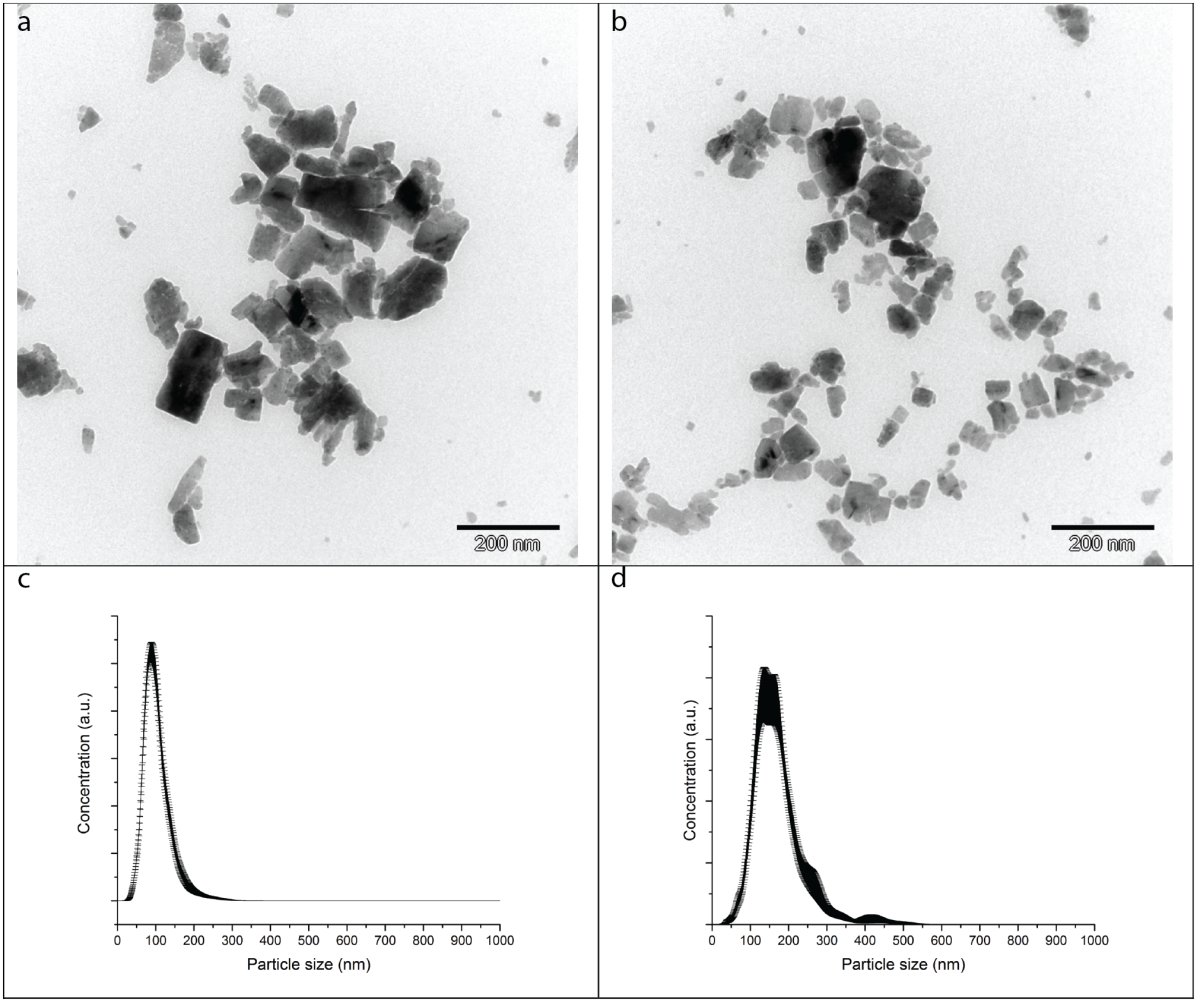
Characterization of Cu particles in the micronized Cu azole (MCA) formulations assessed. TEM micrographs of (a) MCA_HTBA [40] and (b) MCA_LTBA; average particle size distribution of (c) MCA_HTBA and (d) MCA_LTBA. Data represented as mean ± standard deviation of seven repetitions. The MCA_HTBA formulation contains high amount of tebuconazole (TBA) (5% w/w), whereas MCA_LTBA contains low amount of TBA (0.4% w/w).

The Cu particles in the two MCA formulations were comparable and appeared heterogeneous in size and morphology, as shown in the TEM micrographs (Fig 1a and 1b). The size distributions were also similar for the MCA formulations (Fig 1c and 1d). The mean diameter was 104 ±1.7 nm (mode: 87±2.2 nm) for MCA_HTBA and 174±5.9 nm (mode: 150±8.2 nm). Therefore, the Cu particles were solely in the nano-range. The Cu particles in diluted MCA_HTBA and MCA_LTBA had an average ζ-potential of -21.0±0.4 mV and -16.5±1.4 mV respectively, indicating suspensions that tend to aggregate.

### Screening for Cu-tolerance

**Fig 2:**
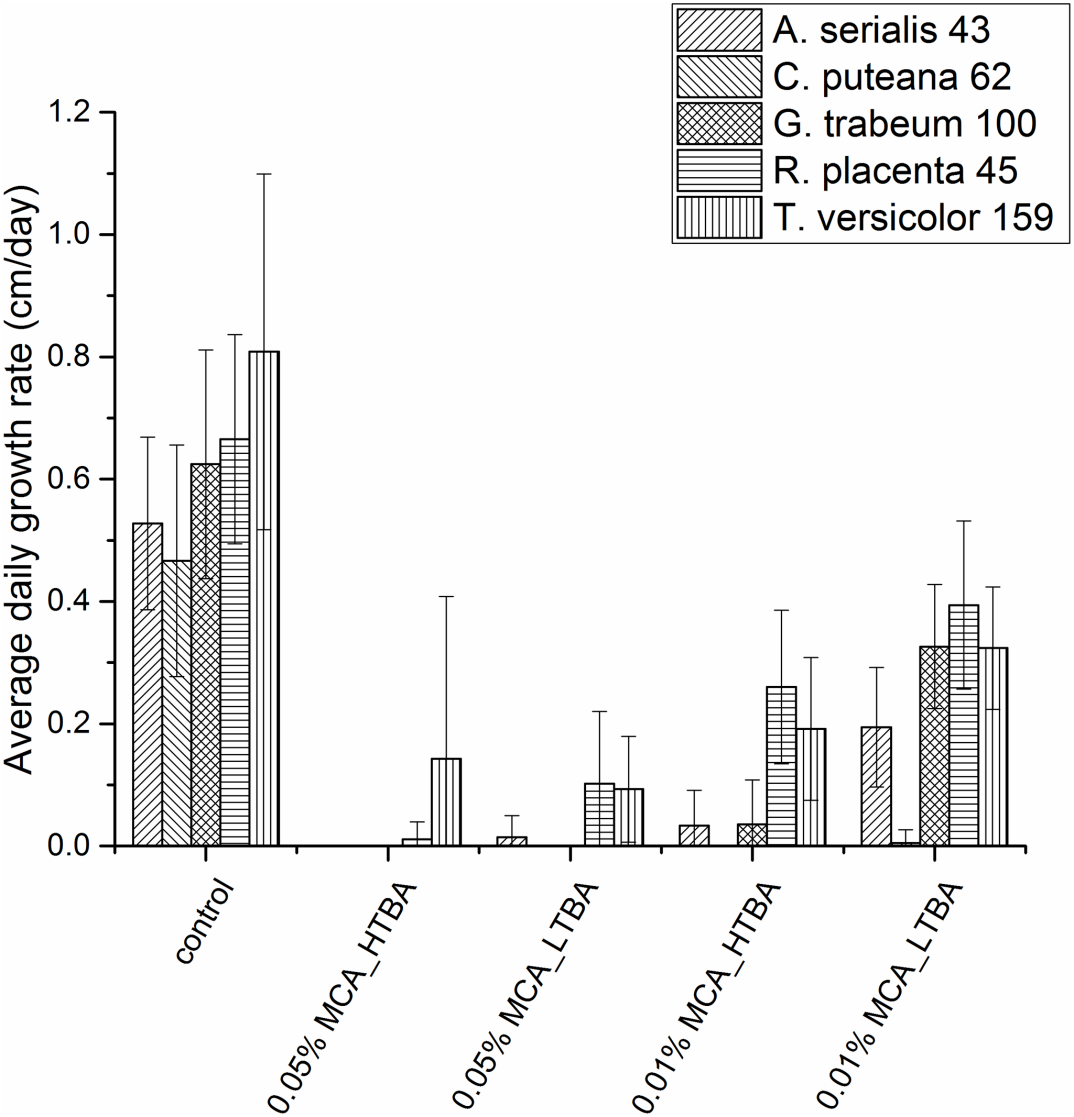
Influence of different concentrations and formulations of micronized copper azole (MCA) on the daily growth rate of *A*. *serialis* 43, *C*. *puteana* 62, *G*. *trabeum* 100, *R*. *placenta* 45, and *T*. *versicolor* 159. The MCA_HTBA formulation contains high amount of TBA (5% w/w), whereas MCA_LTBA contains low amount of TBA (0.4% w/w). Data represented as mean ± standard deviation of three repetitions.

We evaluated the growth of different wood-destroying fungi in MCA-amended media to identify the most Cu-tolerant strain for subsequent studies. Fig 2 shows the mean growth rate for *A*. *serialis* 43, *C*. *puteana* 62, *G*. *trabeum* 100, *R*. *placenta* 45, and *T*. *versicolor* 159 in the different media.

Both concentrations of the MCA formulations caused appreciable reductions in fungal growth rate compared to the controls (p-value < 0.001). *C*. *puteana* 62 was not able to grow in any of the amended media, showing no tolerance to Cu or TBA, whereas *A*. *serialis* 43 and *G*. *trabeum* 100 effectively grew only in 0.01% MCA_LTBA. Differences in overall fungal growth rates were more evident at 0.01% for MCA_LTBA and MCA_HTBA, as distinct patterns were apparent (p-value < 0.001). Media with 0.05% MCA similarly inhibited fungal growth. Minor growth rates at such concentrations were recorded only for *T*. *versicolor* 159 and *R*. *placenta 45*. These two strains also outperformed the other fungi at lower concentrations (p-value < 0.001). In particular, mean growth of *R*. *placenta* 45 was overall the highest, which indicated a high Cu-tolerance. Therefore, this strain was selected for the subsequent tests.

### Fungal response to Cu^2+^ ions and particles

**Fig 3:**
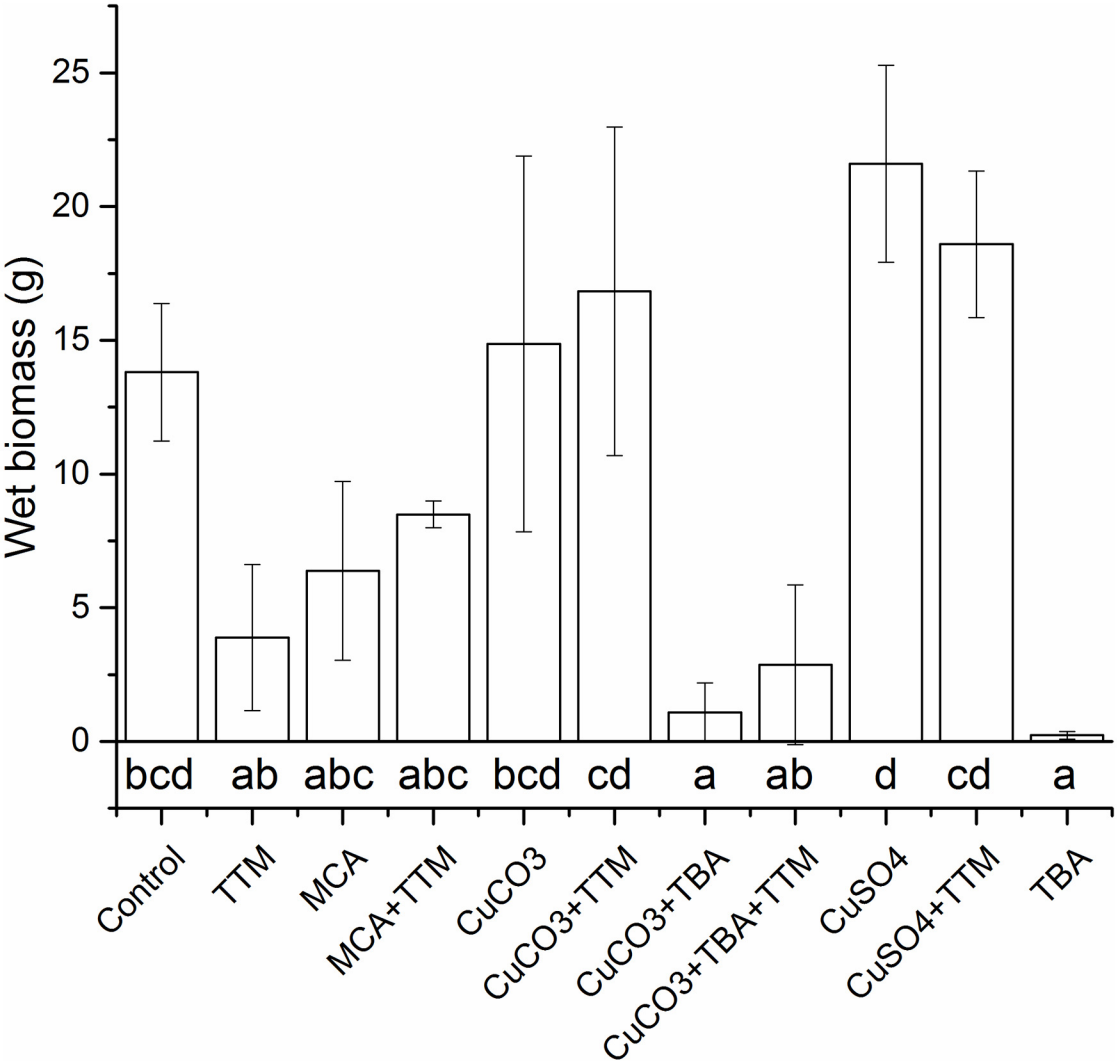
Influence of different forms of Cu on the fungal wet biomass produced by *R*. *placenta* 45 in liquid cultures. TTM = 0.02 mM ammonium tetrathiomolybdate; MCA = 0.02 mM of free Cu^2+^ ions in micronized copper azole; CuCO3 = 0.02 mM Cu carbonate; TBA = 5% w/w tebuconazole; CuSO4 = 0.02 mM Cu sulfate. Data represented as mean ± standard deviation of three repetitions. Shared letters indicate no significant difference in wet biomass production, different letters denote significant differences in wet biomass production after the Tukey’s HSD test.

We assessed the response of *R*. *placenta* 45 to Cu ions, NPs or bulk material to determine if nano effects may account for a superior efficacy of MCA. The effect of Cu^2+^ from dissolution of CuSO_4_, nano Cu from MCA, bulk CuCO_3_·Cu(OH)_2_, and TBA on fungal growth is shown in Fig 3 as mean fungal wet biomass values (dried biomass are in accordance and are not shown).

The differences observed between the tested groups were significant, as indicated by Tukey’s test on the wet biomass production. The addition of TTM to liquid cultures substantially reduced biomass production. What emerged is that TBA strongly suppresses growth of *R*. *placenta* 45. When TBA was associated with Cu (in MCA or with CuCO_3_·Cu(OH)_2_) its inhibition was reduced as follows: TBA > TBA + CuCO_3_·Cu(OH)_2_ > MCA.

#### Laccase activity

Laccase was detected in both MCA- treated (MCA_LTBA and MCA_HTBA) and untreated wood. The amount detected was minor (approx. 1U/L). On the other hand, monitoring of the enzymatic reaction of laccase in liquid cultures by spectrophotometry did not indicate the production of laccase by *R*. *placenta* 45 in the growth media after 2, 4 and 9 weeks incubation period (data available through the ETH Data Archive at: http://doi.org/10.5905/ethz-1007-21).

#### Oxalic acid production

**Fig 4:**
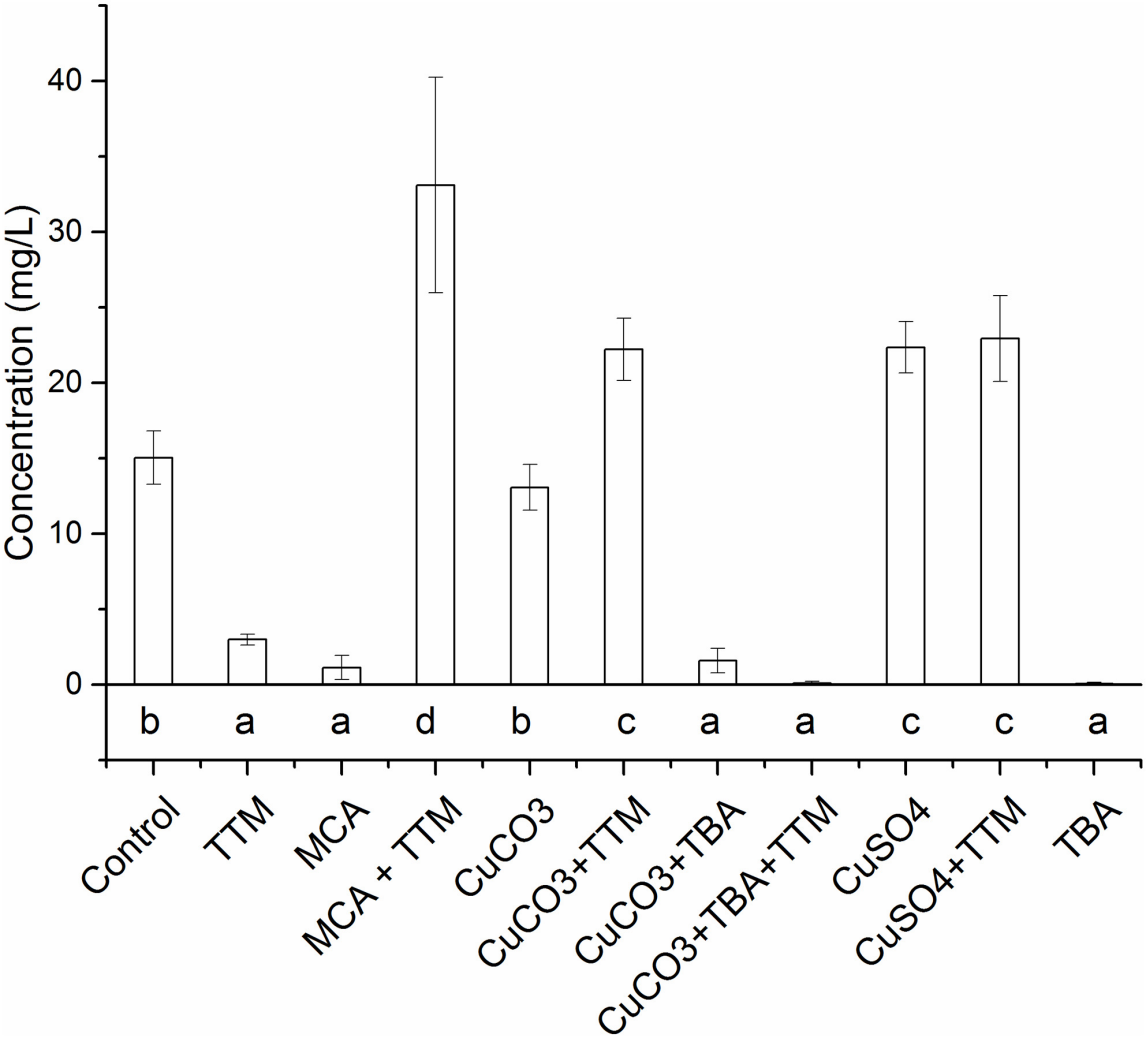
Influence of different forms of Cu on the oxalic acid production by *R*. *placenta* 45 in liquid cultures. TTM = 0.02 mM ammonium tetrathiomolybdate; MCA = 0.02 mM of free Cu^2+^ ions in micronized copper azole; CuCO3 = 0.02 mM copper carbonate; TBA = 5% w/w tebuconazole; CuSO4 = 0.02 mM Cu sulfate. Data represented as mean ± standard deviation of three repetitions. Shared letters indicate treatments that were not significantly different, different letters denote significant differences in treatments after the Tukey’s HSD test.

Fig 4 shows the mean values of oxalic acid produced by *R*. *placenta* 45 at different conditions.

The amount of oxalic acid measured represents only soluble free acid and salts, but it does not take into account the copper oxalate and/or calcium oxalate water insoluble precipitates. The differences observed between the different groups were significant (Tukey’s test). Similarly to the results highlighted in the fungal biomass tests, TBA heavily suppressed the production of oxalic acid by *R*. *placenta* 45. Oxalic acid production in cultures with MCA, CuCO_3_·Cu(OH)_2_, CuCO_3_·Cu(OH)_2_+TBA was lower than in the controls. The addition of TTM to the Cu- amended liquid cultures resulted in an increase in the oxalic acid production from MCA and CuCO_3_·Cu(OH)_2_, both containing CuCO_3_·Cu(OH)_2_, whereas it did not cause any major difference in CuCO_3_·Cu(OH)_2_+TBA and CuSO_4_. The highest oxalic acid concentration was measured in cultures exposed to MCA + TTM.

The protein profiles obtained by SDS-PAGE showed no difference in the protein expression profiles involved for *R*. *placenta* 45 under different growth conditions (data available through the ETH Data Archive at: http://doi.org/10.5905/ethz-1007-21), therefore it is evident that laccase and oxalic acid are the main contributors and no further protein analysis to determine different behavior due to Cu exposure was performed.

### Efficacy of MCA active substances

**Fig 5:**
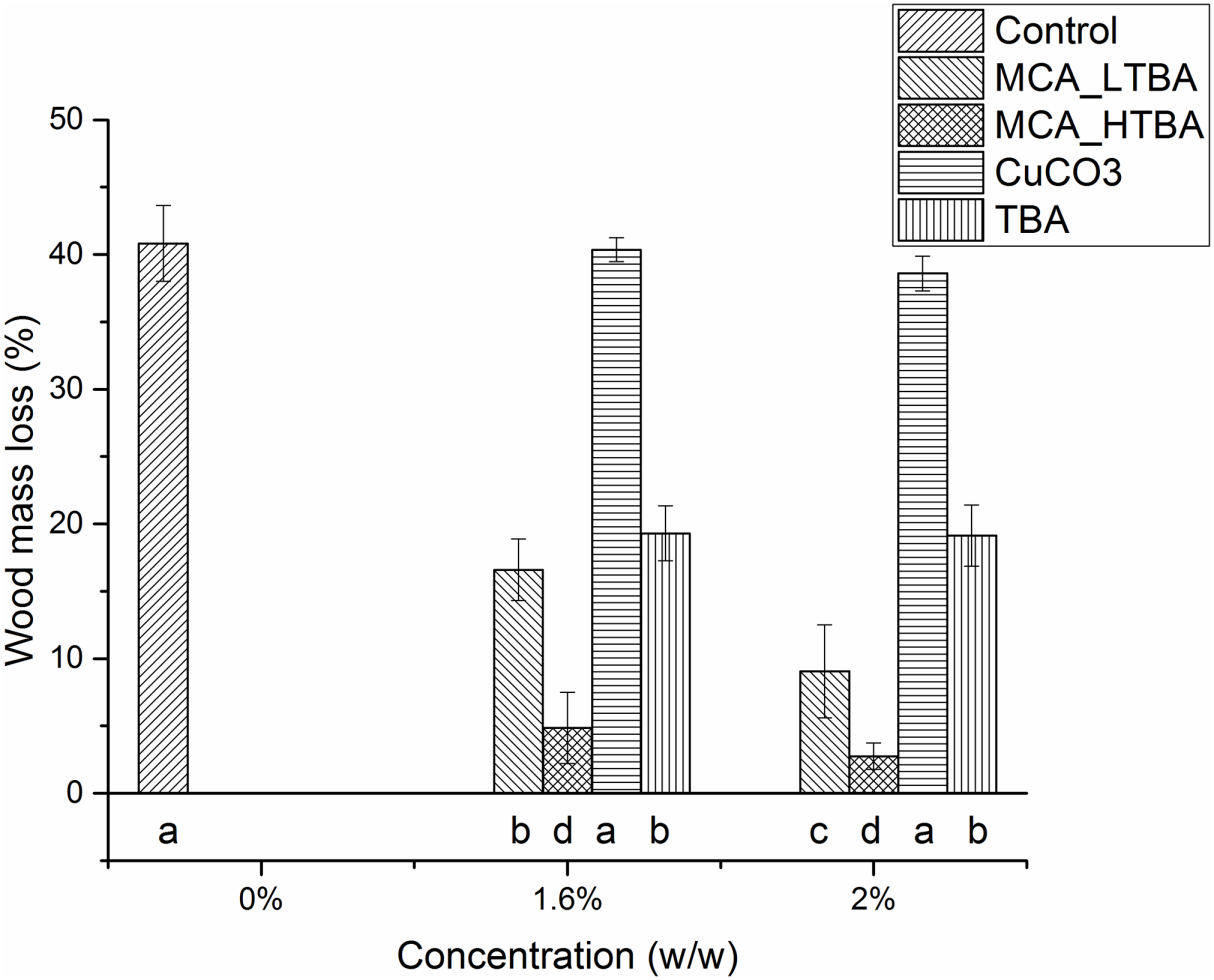
Assessment of micronized Cu azole (MCA) formulations, associated mass losses and the role of each active substance for wood protection against *R*. *placenta* 45. CuCO3 = Cu carbonate; TBA = tebuconazole. The MCA_HTBA formulation contains a high amount of TBA (5% w/w), whereas MCA_LTBA contains a low amount of TBA (0.4% w/w). Data are represented as mean ± standard deviation of four repetitions. Shared letters indicate treatments that were not significantly different, different letters denote significant differences in treatments after the Tukey’s HSD test.

We assessed the contribution of TBA and CuCO_3_·Cu(OH)_2_ in MCA-treated wood. Wood mass losses for the different treatments are presented in Fig 5.

MCA_HTBA, MCA_LTBA and TBA effectively protected wood against fungal colonization and degradation. In particular, MCA_HTBA and MCA_LTBA significantly inhibited fungal growth even at the lowest concentration of 0.8% compared to the controls (p-value < 0.001 in both cases, data available through the ETH Data Archive at: http://doi.org/10.5905/ethz-1007-21). Wood samples treated with CuCO_3_·Cu(OH)_2_ did not reduce mass losses (p-value = 1 for both concentrations). The effects of MCA_HTBA and MCA_LTBA were significantly different at concentrations ≥ 1.33% (data available through the ETH Data Archive at: http://doi.org/10.5905/ethz-1007-21): while the mass losses of MCA_HTBA-treated wood samples were < 3%, mean mass losses of MCA_LTBA-treated wood samples decreased by 9.1%. At concentrations of 1.6% the effect of MCA_LTBA was comparable to the equivalent amount of TBA alone (p-value = 0.997), whereas at concentrations of 2% MCA_LTBA showed a better performance (p-value < 0.001).

## Discussion

Cu is currently used to protect wood from fungal decomposition due to its antifungal properties. In particular, Cu is responsible for interference with homeostatic processes and cell membrane functions [41], protein and enzyme damage and precipitation [42], production of reactive oxygen species [43, 44] and DNA disruption [45]. When Cu is available as NPs these effects may be enhanced. We investigated here in a systematic approach which formulation (ionic, nano or bulk) of Cu is the most effective against Cu-tolerant basidiomycetes. We discriminated between the effects caused by the particles themselves and those caused by their dissolution into Cu^2+^ ions using TTM, a chelator for Cu^2+^ ions.

Our results showed that *T*. *versicolor* 159 and *R*. *placenta* 45 were the two strains that were less influenced by the MCA formulations. We mainly attributed this behavior to TBA-tolerance mechanisms for the white rot fungus *T*. *versicolor* 159 [46], and to Cu-tolerance mechanisms for the brown rot fungus *R*. *placenta* 45 [47]. The main mode of action for TBA consists in fungal cell membrane disruption by inhibition of ergosterol formation [48], whereas Cu exerts its toxic effects on fungal cells by disrupting different basic metabolic processes. Therefore, *R*. *placenta* 45 was selected for the subsequent studies, as it would provide an indication for possible nano effects exerted by MCA on highly Cu-tolerant fungi that would reduce the Cu threshold level and would result in effective protection of wood at lower Cu concentration than the Cu^2+^ ion or bulk counterpart.

The liquid culture study confirmed the suitability of TTM to discriminate between Cu^2+^ ionic and particle effects. In TTM only-amended cultures, biomass and oxalic acid production were lower than in the control cultures, indicating that TTM bound to essential Cu^2+^. Therefore, the study shows that TTM can be effectively employed for studies on Cu-based NPs, for instance in the field of nanotoxicology, where a similar approach has been developed for zinc oxide NPs by Bürki-Thurnherr et al. [49].

It was not possible to identify the presence of laccase in liquid cultures, although this was clearly detected on wood in the EN 113 study. In addition, the amount of laccase detected in untreated and MCA-treated wood was similar. These two findings indicate that laccase is probably not the principal mechanism for Cu detoxification in *R*. *placenta*. Furthermore, we have evidence that supports the fundamental role played by laccase in the Fenton reaction. The Fenton reaction is used by brown and white rot fungi to initialize the attack of wood, as it allows the depolymerization of polysaccharides and lignin by generating radicals [50], whereas in artificial media sugars are readily available, hence radicals are not required. Fungal biomass and oxalic production measurements provided a clear picture on fungal response to Cu in its various forms and TBA. The lower oxalic acid concentrations found in MCA, CuCO_3_·Cu(OH)_2_, CuCO_3_·Cu(OH)_2_+TBA cultures is in good agreement with Green and Clausen findings [51], which revealed that the oxalic acid production of two *R*. *placenta* strains was reduced in Cu-treated wood. The higher oxalic acid concentrations in CuSO_4_ (Cu^2+^ ions)-amended cultures can be related to the increased biomass produced. In addition, the oxalic acid production was stimulated in Cu+TTM-amended cultures, therefore confirming that free Cu can reduce oxalic acid production. For both fungal biomass and oxalic acid production, the major inhibiting agent was TBA, however the effects were reduced in the presence of Cu, especially for MCA. Therefore, we hypothesize that Cu, here at sub-lethal concentrations, can stimulate growth and enzyme production of *R*. *placenta*, as indicated in former studies [52, 53]. In addition, for MCA other chemicals in the formulation may have influenced fungal growth by providing additional nutrients. Thus, for the concentrations used, there was no indication of a Cu+TBA additive or synergistic effects against Cu-tolerant fungi. Although there is a lack of scientific literature on Cu and TBA additive, synergistic or antagonist effects, Sun et al. [54] showed a similar behavior for a range of moulds that can biotransform TBA [55, 56], hence effectiveness of pure Cu was higher than Cu combined with TBA. In any case, we found no evidence for a specific nano effect against *R*. *placenta* and the main active substance against *R*. *placenta* was clearly TBA. This means that the fungus is able to recognize Cu also as MCA NPs and can trigger the same Cu-tolerance mechanisms typically shown in the presence of bulk Cu or Cu ions. No additional protein expression patterns were evident in SDS-PAGE analysis, therefore oxalic acid and laccase were valid parameters for determining fungal response to Cu.

Finally, to complete the study, we investigated fungal growth on treated wood i.e. a more natural setting. In this case, the EN 113 guidelines were applied and mass losses of wood treated with bulk CuCO_3_·Cu(OH)_2_, TBA and two MCA formulations differing in TBA content were compared. This test provides indications on the expected short term effectiveness of the wood treatments.

The recorded mass losses were in good agreement with our findings on fungal growth in liquid cultures. Even in treated wood TBA is largely responsible for the effectiveness of MCA, although high concentrations of Cu also affect the performance. In particular, for the formulations and wood decay fungi assessed, we propose a 1.6% MCA < Cu threshold ≤ 2% MCA. The low effectiveness of CuCO_3_·Cu(OH)_2_ is mainly attributed to the poor penetration into the wood: the wood samples did not show any color change towards green/blue due to the presence of Cu, and unreacted CuCO_3_·Cu(OH)_2_ only appeared as fine dust unbound on the sample surfaces.

In conclusion, the NPs in the MCA formulations assessed did not provide additional protection against *R*. *placenta* and the main effectiveness has to be attributed to TBA. Therefore, considering the antifungal properties, the efficacy of the MCA formulations tested are not better than conventional Cu azole formulations that do not employ nanotechnologies. MCA-treated wood will still be susceptible to biodegradation by Cu- or TBA-tolerant fungi. From a life cycle assessment perspective, MCA is less eco-efficient than Cu azole, due to the higher energy consumption during the milling process of MC [57]. However, this would also imply no additional risk for the microbial community in vicinity of MCA-treated wood.

Further studies with other wood-destroying fungi and different MC formulations are required to provide a more comprehensive picture on MC NPs effects on wood-destroying fungi. In addition, field studies are required to confirm our lab-scale findings and assess the long term performance. In particular, TTM could not be used in the EN 113 test, due to the absence of a liquid environment that would allow chelation of Cu^2 +^ ion. Therefore, tests with wood samples immersed in liquid cultures and TTM, in a setting similar to the one suggested by the EN 275 [58] guidelines, may provide further details on the fungal response to Cu^2+^ ions and particles. Future studies should focus on the fungal gene pathways that are involved for tolerance mechanisms against TBA and Cu.
